# Movement behaviour patterns in patients with hip and/or knee osteoarthritis in the physical therapy setting: a cross-sectional study

**DOI:** 10.1186/s12891-020-03644-0

**Published:** 2020-10-06

**Authors:** Anne Maria Sjoerdtje de Hoop, Corelien Jacoba Johanna Kloek, Martijn Frits Pisters, Cindy Veenhof

**Affiliations:** 1 Department of Rehabilitation, Physiotherapy Science & Sport, UMC Utrecht Brain Center, University Medical Center Utrecht, Utrecht University, Utrecht, The Netherlands; 2 Research Centre for Healthy and Sustainable Living, Research group Innovation of Movement Care, University of Applied Sciences Utrecht, Utrecht, The Netherlands; 3 Center for Physical Therapy Research and Innovation in Primary Care, Julius Health Care Centers, Utrecht, The Netherlands; 4 Department of Health Innovations and Technology, Fontys University of Applied Sciences, Eindhoven, The Netherlands

**Keywords:** Osteoarthritis, Physical activity, Sedentary behaviour, Movement behaviour

## Abstract

**Background:**

Osteoarthritis is one of the most common chronic joint diseases, mostly affecting the knee or hip through pain, joint stiffness and decreased physical functioning in daily life. Regular physical activity (PA) can help preserve and improve physical functioning and reduce pain in patients with osteoarthritis. Interventions aiming to improve movement behaviour can be optimized by tailoring them to a patients’ starting point; their current movement behaviour. Movement behaviour needs to be assessed in its full complexity, and therefore a multidimensional description is needed.

**Objectives:**

The aim of this study was to identify subgroups based on movement behaviour patterns in patients with hip and/or knee osteoarthritis who are eligible for a PA intervention. Second, differences between subgroups regarding Body Mass Index, sex, age, physical functioning, comorbidities, fatigue and pain were determined between subgroups.

**Methods:**

Baseline data of the clinical trial ‘e-Exercise Osteoarthritis’, collected in Dutch primary care physical therapy practices were analysed. Movement behaviour was assessed with ActiGraph GT3X and GT3X+ accelerometers. Groups with similar patterns were identified using a hierarchical cluster analysis, including six clustering variables indicating total time in and distribution of PA and sedentary behaviours. Differences in clinical characteristics between groups were assessed via Kruskall Wallis and Chi^2^ tests.

**Results:**

Accelerometer data, including all daily activities during 3 to 5 subsequent days, of 182 patients (average age 63 years) with hip and/or knee osteoarthritis were analysed. Four patterns were identified: inactive & sedentary, prolonged sedentary, light active and active. Physical functioning was less impaired in the group with the active pattern compared to the inactive & sedentary pattern. The group with the prolonged sedentary pattern experienced lower levels of pain and fatigue and higher levels of physical functioning compared to the light active and compared to the inactive & sedentary.

**Conclusions:**

Four subgroups with substantially different movement behaviour patterns and clinical characteristics can be identified in patients with osteoarthritis of the hip and/or knee. Knowledge about these subgroups can be used to personalize future movement behaviour interventions for this population.

**Trial registration:**

Dutch clinical trial registration number of e-Exercise Osteoarthritis: NTR4224.

## Background

The incidence of osteoarthritis is increasing and is expected to continue so in the following years. This increase has been associated with an aging population and rising obesity numbers, which are risk factors for the development of the disease [1, 2]. Symptoms of osteoarthritis such as pain and decreased physical functioning result in high amounts of medical visits and extensive use of medication, which leads to high healthcare costs both short and longer term [3].

Regular physical activity (PA) preserves and improves physical functioning and reduces pain symptoms in patients with osteoarthritis [4, 5]. Conversely, spending more time sedentary is associated with increased functional limitations [6]. Especially sedentary periods of over 30 subsequent minutes should be avoided [7, 8]. However, PA levels are often lower in patients with osteoarthritis compared to healthy adults [9, 10]. Following recent guidelines by the World Health Organization (WHO), adults should perform moderate to vigorous PA for at least 150 min per week in bouts of at least 10 min [11]. Results showed that fewer than one out of seven men and one out of twelve women with knee osteoarthritis meet these guidelines [10]. Factors associated with these decreased PA levels include older age, higher Body Mass Index (BMI), lower physical functioning and comorbidity [12, 13].

PA is often assessed with either a single parameter or multiple independent parameters, such as time spent in PA, the intensity of performed activities, or amount of active periods in a certain intensity (also called bouts) per day [14]. However, when taking only a single parameter into account, a lot of relevant information about movement behaviour is ignored. Accelerometers, such as the ActiGraph GT3X, can assess PA in different dimensions. For instance, one can assess the distribution of PA by measuring both the amount of active bouts and the total time spent in PA. Additionally, this can be obtained for PA in different intensities and for sedentary behaviours. There is large variation in how people score across all dimensions [15]. Because of this heterogeneity, the Sedentary Behaviour Research Network recently advocated a multifaceted definition of ‘movement and non-movement behaviours’, including all dimensions of PA, sedentary behaviour and sleep [16]. Accordingly, assessing movement behaviour in its full complexity should be preferred over single measures of PA.

Interventions for patients with osteoarthritis often aim to improve movement behaviour. Even though these interventions have yielded positive results, effects are small and largely inconsequential for the longer term [17]. The effectiveness of these interventions can be improved by tailoring them to patients’ personal characteristics and preferences [18]. Second, often only one dimension of movement behaviour, such as time spent in PA, is adressed [19] while different dimensions should be targeted simultaneously in order to yield the best results [15]. This means that understanding behaviour in its complete context is needed in order to know where best to intervene and how [20]. It can be assumed that a patient’s starting point - their current movement behaviour including all dimensions, e.g. their movement behaviour pattern - can provide direction for treatment.

Before health practitioners are able to measure and interpret multidimensional movement behaviour, the heterogeneity in movement behaviour of patients with osteoarthritis should be explored. With statistical methods such as cluster analysis, individuals can be grouped, based on similarities in multiple movement behaviour dimensions simultaneously. Recent research has identified patterns in movement behaviours of patients with COPD, chronic cancer-related fatigue and hemophilia [21–23]. It has been argued that persons with different movement behaviour patterns should have substantially different treatment goals [21, 22].

Exploring the differences between subgroups regarding clinical characteristics provides further insight into the characteristics and common problems of specific subgroups. This especially holds for factors that have been associated with decreased PA levels, such as physical functioning and comorbidity. Detailed, multidimensional descriptions of movement behaviour in patients with osteoarthritis of the hip and/or knee, as well as patient characteristics related to these behaviours, are lacking in current literature.

Therefore, this study aimed to identify subgroups based on movement behaviour patterns in patients with osteoarthritis of the hip and/or knee who are eligible for a PA intervention. The second aim was to determine the differences between subgroups regarding BMI, sex, age, physical functioning, comorbidities, fatigue and pain.

## Materials and methods

### Study design and setting

In this cross-sectional study, baseline data from the multicentre prospective trial ‘e-Exercise Osteoarthritis’ [24] were analysed. For the e-Exercise Osteoarthritis trial, 208 participants were recruited in 143 primary care physical therapy practices in three provinces in the Netherlands. Data was collected between September 2014 and May 2015. A more complete description of the recruitment can be found in the protocol article [24].

### Participants

Participants were eligible for inclusion in the trial e-Exercise Osteoarthritis if they were aged between 40 and 80 years and were diagnosed with hip and/or knee osteoarthritis according to the clinical criteria of the American College of Rheumatology [25]. Excluded were patients who (a) were on a waiting list for a hip or knee replacement surgery, (b) had a contra-indication for physical activity without supervision, determined with the Physical Activity Readiness Questionnaire (PAR-Q), (c) had a self-reported physically active lifestyle according to the WHO health norm for PA [11], (d) participated in a physical therapy and/or physical activity program in the last 6 months, (e) had no access to the internet, or (f) were unable to understand the Dutch language.

Patients were included in the current study if accelerometer data from at least three consecutive days of 8 h was available. This is in accordance with guidelines for accurate measuring of physical activity from accelerometer output [26].

### Measures

Movement behaviour was assessed with the GT3X or GT3X+ tri-axial accelerometer (ActiGraph LLC, Pensacola, FL, USA). All participants received the accelerometer with written instructions at baseline and were asked to wear the device for 5 consecutive days, with exception of sleep, showering and swimming, on an elastic belt around their waist. Participants were asked to document all time slots of ‘wear-time’ and ‘non-wear time’ in a diary. The device did not provide the participants with feedback regarding performed movement behaviour.

The raw output of the accelerometer provides three dimensional activity counts per minute and a vector magnitude derived from combined activity counts from the three axes. Activity counts are derived from the frequency and intensity of accelerations and indicate intensity of performed activity, where higher counts stand for higher intensity. Accelerometer counts of the GT3X+ are found appropriate for quantifying activity [27]. There is strong agreement between the activity counts of the GT3X and GT3X+ [28]. Raw accelerometer output was extracted and translated into movement behaviour variables on the computer, with ActiLife software (version 5.6.1; ActiGraph LLC, Fort Walton Beach, FL, USA). Wear-time was automatically assessed by the same software, applying Troiano’s (2007) definition [29]; a minimum length of 60 min and spike level to stop of 100 counts per minute. The resulting periods of wear-time were checked manually based on written diaries of the participants, and corrected when necessary.

Extracted variables - see also Table 2 - included total minutes spent in moderate to very vigorous (MV) intensity, sedentary and prolonged sedentary activity per day. Furthermore, the variables ‘number of MV bouts per day’, ‘average length of MV bouts’ and ‘number of sedentary bouts per day’ were assessed to measure the distribution of physical activity and sedentary behaviour over the day. The minimum bout length of all variables was 2 min. One exception was the variable ‘total time in sedentary bouts’ with a minimum bout length of 30 min [7, 8]. The minimum bout length of ‘total time spent in MV activity’ was ten subsequent minutes, in line with the specification of MV activity in the health norm for PA [11]. MV activity was defined as continuous activity of at least 2690 counts/minute [30] and sedentary behaviour was defined as continuous activity of less than 100 cpm [31, 32].

Physical functioning was assessed with the subscale ‘function in daily living’ of the Hip Osteoarthritis Outcome Score (HOOS) [33] or the Knee injury and Osteoarthritis Outcome Score (KOOS) [34]. This subscale is scored on a 5-point Likert scale where 0 stands for extreme symptoms/problems and 4 stands for no symptoms/problems. The average score of all items was normalized to a score ranging from 0 to 100. Patients diagnosed with both hip and knee osteoarthritis filled in both questionnaires and the lowest score was used for analyses.

Weight (in kg), length (in meters), age, sex and comorbidities were assessed in the baseline questionnaire. BMI was categorized in (a) < 25 kg/m^2^ (normal weight), (b) 25–29.9 kg/m^2^ (overweight) and (c) > 30 kg/m^2^ (obese). Comorbidities were categorized to (a) none, (b) one, or (c) two or more. Pain and tiredness were assessed on a numeric rating scale, whereby 0 stood for no pain/ not tired and 10 indicated the worst imaginable pain/ very tired [35, 36].

### Statistical methods

Statistical analyses were performed with IBM SPSS Statistics for Windows (version 24, IBM Corp., Armonk, N.Y., USA). Prior to the statistical analyses, the data was checked for missing values and outliers. Correlations between clustering variables were assessed to check for multicollinearity. A Pearson’s correlation coefficient r > .90 was deemed problematic for the clustering procedure.

A hierarchical cluster analysis following Ward’s linkage method with Euclidean distances was performed. Clustering variables included the movement behaviour variables (Table 1). Based on visual inspection of the dendrogram and interpretability of the clusters, a choice was made for a number of clusters (subgroups) that described the distinct movement behaviours best. Because the data within the identified subgroups was not normally distributed, a Kruskall Wallis test was performed to determine if subgroups were significantly different on all clustering variables. In the secondary analysis, Chi^2^ tests (for nominal variables) and a Kruskall Wallis test (for variables at interval and ratio level) were performed to assess the differences between the subgroups regarding BMI, sex, age, physical functioning, comorbidities, tiredness and pain.

**Table 1:** Participant characteristics

Variable	Category	Included participants (*n* = 182)		Excluded participants (*n* = 26)	
Age in years, mean (SD)	.	63.0	(8.6)	63.4	(10.0)
Sex, n (%)	Male	60	(33.0)	7	(26.9)
	Female	122	(67.0)	19	(73.1)
Body Mass Index in kg/m^2^, n (%)	< 25	59	(32.4)	3	(11.5)
	25–29.9	76	(41.8)	10	(38.5)
	> 30	47	(25.8)	13	(50.0)
Location of osteoarthritis, n (%)	Knee	119	(65.4)	19	(73.1)
	Hip	35	(19.2)	3	(11.5)
	Both	28	(15.4)	4	(15.4)
Time since diagnosis, n (%)	< 1 year	39	(21.4)	2	(7.7)
	1 to 5 years	69	(37.9)	11	(42.3)
	> 5 years	74	(40.7)	13	(50.0)
Comorbidities, n (%)	None	111	(61.0)	13	(50.0)
	Single	33	(18.1)	7	(26.9)
	Multiple	38	(20.9)	6	(23.1)
Physical functioning [0–100], mean (SD)	.	58.5	(19.7)	58.8	(22.4)
Fatigue [0–10], mean (SD)	.	5.1	(2.7)	5.0	(3.4)
Pain [0–10], mean (SD)	.	5.3	(2.3)	5.6	(2.4)
Accordance with health norm^a^, n (%)	.	28	(15.4)	–	–

*SD* standard deviation, *n* number of individuals, […] = scale range

^a^: health norm for physical activity, specified as 150 min of MV activity per week in bouts of at least 10 minutes [11]

### Sample size

There are no strict guidelines to determine the required sample size for a cluster analysis. The rule-of-thumb *n* ≥ 2^m^, where m stands for the amount of clustering variables, is generally accepted [37]. For this study, this leads to a minimum sample size of *n* = 2^6^ = 64. The number of participants in the sample was therefore sufficient.

## Results

One participant was excluded because the accelerometer data were invalid due to incorrect settings. Twenty-four participants were excluded because less than 3 days of 8 h of accelerometer data were recorded. One outlier was excluded because one outcome was distanced 6.7 standard deviations from the mean, and therefore it severely influenced the clustering procedure. Finally, 182 participants were included in this study. Characteristics of in- and excluded participants are shown in Table 1. Some minor differences exist between in- and excluded participants but there was no strong indication for sampling bias. In Table 2, total group means on all clustering variables are shown. In total, data of 122 females and 60 males, with an average age of 63.0 years (sd = 8.6 years), were analysed. All correlation coefficients between clustering variables were within acceptable limits, defined as Pearson’s r < .9.

**Table 2:** Clustering variables and group means

Variable name	Minimum bout length in minutes	VM3 counts/min	Total group mean (SD)	
**Total hours in sedentary behaviour per day**	2	< 100	6.2	(1.5)
**Total hours in prolonged sedentary behaviour per day**	30	< 100	2.2	(1.4)
**Number of sedentary bouts per day**	2	< 100	53.2	(10.0)
**Total minutes in MV activity per day**	10	≥ 2690	10.0	(14.3)
**Number of MV bouts per day**	2	≥ 2690	5.3	(4.6)
**Average length (minutes) of MV bouts**	2	≥ 2690	3.8	(2.0)

*MV* moderate to very vigorous, *VM3* triaxial vector magnitude

### Identification of movement behaviour patterns

**Fig. 1:**
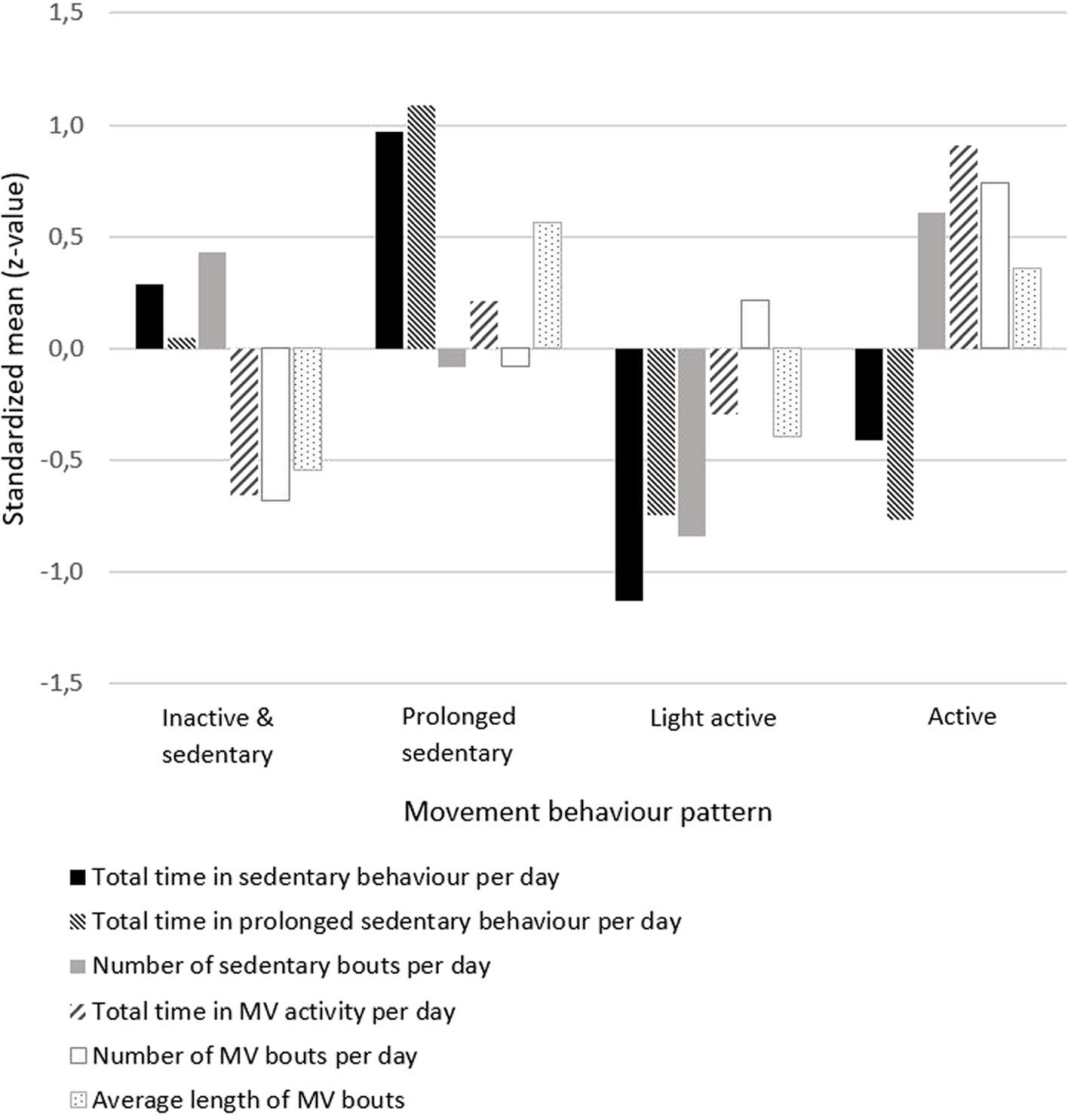
Standardized group means on clustering variables

Table 3 shows the mean scores on the clustering variables and Fig. 1 shows the normalized mean outcomes per subgroup. The dendrogram, illustrating the clustering procedure, can be found in Additional file 1. All clustering variables contributed significantly to the identification of clusters (*p* < .001).

**Table 3:** Mean scores of pattern groups on clustering variables (*N* = 182)

	*Inactive & sedentary pattern*	*Prolonged sedentary pattern*	*Light active pattern*	*Active pattern*
	*n* = 47	*n* = 54	*n* = 45	*n* = 36
**Total hours in sedentary bouts**^**a**^ **per day** (SD)	6.7 (0.8)	7.7 (1.1)	4.5 (0.9)	5.6 (0.7)
**Total hours in prolonged sedentary bouts**^**b**^ **per day** (SD)	2.3 (0.7)	3.7 (1.1)	1.2 (0.7)	1.1 (0.7)
**Number of sedentary bouts**^**a**^ **per day** (SD)	57.5 (6.9)	52.3 (9.2)	44.7 (8.5)	59.3 (9.2)
**Total minutes in MV bouts**^**c**^ **per day** (SD)	0.7 (1.5)	13.0 (11.7)	5.9 (6.1)	23.0 (22.0)
**Number of MV bouts**^**a**^ **per day** (SD)	2.1 (1.7)	4.9 (3.5)	6.2 (3.7)	8.7 (6.5)
**Average length in minutes of MV bouts**^**a**^ (SD)	2.8 (1.0)	5.0 (2.6)	3.1 (0.9)	4.5 (1.6)

*SD* standard deviation, *n* number of individuals

^a^: minimum bout length: 2 subsequent minutes; ^b^: minimum bout length: 30 subsequent minutes; ^c^: minimum bout length: 10 subsequent minutes

The first pattern was called *inactive & sedentary*. Firstly because the average time spent in MV bouts was close to zero and the number and average length of MV bouts per day was lower compared to the other patterns. Second, the time spent in sedentary bouts and prolonged sedentary bouts, and the number of sedentary bouts were all above the total group average.

The group with the second pattern spent more minutes in sedentary bouts and prolonged sedentary bouts compared to all other patterns. At the same time, the number of sedentary bouts was lower compared to that of the inactive & sedentary, indicating fewer breaks in sedentary time. This pattern was therefore named *prolonged sedentary.* The group with this pattern spent more time in MV activity compared to the inactive & sedentary and the third pattern (light active) but less compared to the fourth (active). This pattern had the highest average length of MV bouts.

The third pattern showed the least minutes in sedentary behaviour and lowest number of sedentary bouts compared to the other patterns. Time spent in prolonged sedentary behaviour was less compared to the inactive & sedentary pattern and to the prolonged sedentary pattern, though comparable with the fourth (active) pattern. The time spent in MV activity and the average duration of MV bouts were below the total group average while the amount of moderate to very vigorous bouts was above average. Likely, this group is mostly active in light intensity activities during the day and therefore the pattern of this group was called *light active*.

The last pattern was characterized by more time in MV activity compared to all other patterns and was called the *active* pattern. Individuals with this pattern on average spent more time in sedentary behaviour compared to people with the light active pattern, though less compared to the prolonged sedentary pattern and the inactive & sedentary pattern. The active pattern showed the most sedentary bouts and the least minutes in prolonged sedentary bouts, indicating more breaks in sedentary time.

### Differences in clinical characteristics between subgroups

Chi^2^ tests identified significant differences between groups regarding sex. Table 4 shows that men were less represented in the groups with the light active pattern and the inactive & sedentary pattern compared to the other groups (*p* < .001). No significant differences were found between pattern groups regarding BMI and comorbidity.

**Table 4:** Differences in clinical characteristics between subgroups

	*Inactive & sedentary pattern*	*Prolonged sedentary pattern*	*Light active pattern*	*Active pattern*
	*n* = 47	*n* = 54	*n* = 45	*n* = 36
**Age**, mean (SD)	63.4 (8.7)	65.1 (8.2)	60.9 (8.3)	62.1 (8.9)
**Physical functioning**, mean (SD)	50.9 (3.0)^a^	67.0 (2.3)	53.6 (2.7)^a^	61.8 (3.1)^b^
**Pain**, mean (SD)	6.0 (0.3)^a^	4.7 (0.3)	5.8 (0.3)	4.8 (0.4)
**Fatigue**, mean (SD)	5.8 (0.4)^a^	4.1 (0.4)	5.7 (0.4)^a^	4.9 (0.4)
**Sex**, n (%)				
Male	6 (12.8)^c^	30 (55.6)^b^	6 (13.3)^c^	18 (50)^b^
Female	41 (87.2)	24 (44,4)	39 (86.7)	18 (50)
**BMI category**, n (%)				
< 25 kg/m^2^	6 (12.8)	18 (33.3)	11 (46.7)	14 (38.9)
25–29.9 kg/m^2^	27 (57.4)	22 (40.7)	13 (28.9)	14 (38.9)
> 30 kg/m^2^	14 (29.8)	14 (25.9)	11 (24.4)	8 (22.2)
**Comorbidities**, n (%)				
None	29 (61.7)	33 (61.1)	27 (60.0)	22 (61.1)
Single	12 (25.5)	8 (14.8)	6 (13.3)	7 (19.4)
Multiple	6 (12.8)	13 (24.1)	12 (26.7)	7 (19.4)

*SD* standard deviation, *n* number of individuals

^a^: significant difference vs. group with prolonged sedentary pattern (*p* < .05); ^b^: significant difference vs. group with inactive & sedentary pattern (*p* < .001); ^c^: significant difference vs. group with prolonged sedentary pattern (*p* < .001)

The Kruskall Wallis test did not indicate significant differences in age between groups. Significant differences were identified for levels of physical functioning, pain and fatigue. Post-hoc tests with Bonferroni correction for multiple testing showed the following:

The group with the prolonged sedentary pattern had a higher level of physical functioning compared to group with the light active pattern (adjusted *p* < .05) and compared to the group with the sedentary non active pattern (adjusted *p* < .001). Second, they had lower pain levels compared to the groups with the inactive & sedentary pattern (adjusted *p* < .05) and the light active pattern. The group with the prolonged sedentary pattern was less fatigued compared to the groups with the light active pattern (adjusted. p < .05) and the inactive & sedentary pattern (adjusted p < .05). The group with the active pattern had significantly higher levels of physical functioning compared to the group with the inactive & sedentary pattern (adjusted. *p* < .001).

## Discussion

The aim of this study was to identify patterns in movement behaviours of patients with osteoarthritis of the hip and/or knee and second, to examine the differences in clinical characteristics between groups with these patterns. The analysis resulted in the distinction of four different movement behaviour patterns: inactive & sedentary, prolonged sedentary, light active and active. Second, differences were found between groups regarding sex, physical functioning, pain and fatigue.

This study highlights the heterogeneity in movement behaviours of patients with hip and/or knee osteoarthritis, resulting in the distinction of four groups with substantially different movement behaviour patterns and clinical characteristics. The results confirm that high levels of sedentary behaviour and high MV activity are not mutually exclusive [21]. For example, the group with the prolonged sedentary pattern spent more minutes in sedentary and prolonged sedentary behaviour compared to the group with the inactive & sedentary pattern, while their daily time in MV activity was also substantially higher. Both being active in MV activity and limiting sedentary time are recommended for a healthy lifestyle and reduced risk of chronic diseases [7, 8, 11]. This example shows that multidimensional measures provide a more complete picture of these behaviours compared to single dimensional measures. Identification of patients’ individual movement behaviour patterns enables the identification of individual goals in movement behaviours.

In order to improve movement behaviour effectively, multiple dimensions should be targeted [15]. It can also be expected that people with different movement behaviour patterns will benefit from different recommendations [21, 22]. For instance, the goal of both persons with the prolonged sedentary pattern and the inactive & sedentary pattern should be to decrease their total sedentary time. At the same time, the inactive & sedentary pattern might need a significant increase in MV activity while for persons with a prolonged sedentary pattern, reducing the length of sedentary bouts could be the second goal. The light active pattern shows a relatively high amount of 2 min-MV bouts while only little time is spent in 10 min-MV bouts. Therefore, lengthening their MV bouts might be an efficient way to improve their movement behaviour. Goals for the active subgroup could be to maintain their high levels of MV activity, while decreasing prolonged sedentary time.

The secondary analyses of this study highlighted other clinically relevant differences in characteristics between groups. First, men were predominantly represented in the groups with the prolonged sedentary pattern and the active pattern. Interestingly, these two groups both spent more time in MV activity compared to the other groups while their sedentary behaviours were dissimilar. These results seem to be in line with an earlier finding that men are more likely to participate in MV activity compared to women [10]. Second, the groups with the light active pattern and the inactive & sedentary pattern both showed higher levels of pain and fatigue and lower physical functioning compared to the other groups. A review by Veenhof et al. pointed out that some studies have found associations between these symptoms and movement behaviour, though results were not consistent [12].

To our knowledge, this is the first study that has identified patterns in movement behaviour of patients with hip and/or knee osteoarthritis. Patterns in movement behaviour have recently been defined in patients with COPD [21] and chronic cancer-related fatigue [22]. Similar to these studies, we identified one most sedentary pattern and one pattern characterized by most MV activity, whereby the latter represents the smallest group. However, where Wolvers et al. described one ‘average’ pattern [22], we identified two pattern with mixed characteristics, i.e. the inactive & sedentary and the light active pattern. Also notable is that the group with the prolonged sedentary pattern spent more minutes in MV activity compared to the inactive & sedentary pattern. In both the two previous studies, spending more time in sedentary behaviour seemed to coincide with less time in MV activity [21, 22]. Unlike these findings, time spent in sedentary behaviour and MV activity appeared more independent in patients with knee and hip osteoarthritis.

Some limitations of this study should be mentioned. First, because the GT3X and GT3X+ are not water resistant, time spent swimming could not be recorded. For the 19 participants who reported a swimming activity, measured activity was therefore underestimated. Furthermore, differences in movement behaviour may exist between week- and weekend days, especially among workers. Unfortunately, we did not manage to control for this in the analysis and this might therefore have influenced the results.

Also, based on best available literature, a choice was made for the cut-points for sedentary behaviour and MV activity. It is known that this choice affects measures of movement behaviour [38]. The data that result from these cut-points are suitable to compare the identified groups, though comparisons between studies using different cut-points should be made with caution.

Notably, based on our data 28 participants (15,4%) met the WHO guidelines for PA, while people meeting this norm in their daily life were excluded from our study. There can be various reasons for this discrepancy between self-reported PA during the anamnesis and PA assessed with the accelerometer. First, participants were aware that their PA was being recorded and this may have led to an increase in daily PA. Another reason may be that it is challenging for physical therapists to estimate daily PA based on self-reported information of the patient. Consequently, the movement behaviour patterns identified in this study apply to this population of individuals with knee and/or hip osteoarthritis in the physical therapy setting. Further research is needed to confirm the presence of these patterns in larger populations.

Another point to consider is that large variances are often seen in movement behaviour measures of patients with hip and knee osteoarthritis [10, 22]. In the current study large standard deviations were also observed for the outcomes on clustering variables in all patterns, which challenges formulation of precise descriptions of patterns. It should therefore be realized that the descriptions of the identified patterns do not comprise small individual differences. Knowledge about existing movement behaviour patterns can be a first step towards more personalized PA interventions, though an individuals’ context and preferences should always be accounted for.

In conclusion, four patterns in movement behaviours of patients with hip and/or knee osteoarthritis were identified. Differences between groups with these patterns exist regarding sex, physical functioning, pain and fatigue. Treatment for patients with osteoarthritis of the hip and/or knee can be personalized by incorporating knowledge on movement behaviour interventions and target the dimensions that will benefit the individual most.

## Supplementary information

**Additional file 1:** Dendrogram.

## Data Availability

The data that support the findings of this study are available on request from the corresponding author AH. The data are not publicly available due to them containing information that could compromise research participant privacy or consent.
