# Supplementary material for: Movement behaviour patterns in patients with hip and/or knee osteoarthritis in the physical therapy setting: a cross-sectional study
Source: BMC Musculoskelet Disord. 2020 Oct 6;21:651. doi: 10.1186/s12891-020-03644-0 (PMC7539450; doi:10.1186/s12891-020-03644-0)
Supplement: Supplementary file 1 — Additional file 1: Dendrogram. [file 12891_2020_3644_MOESM1_ESM.pdf]

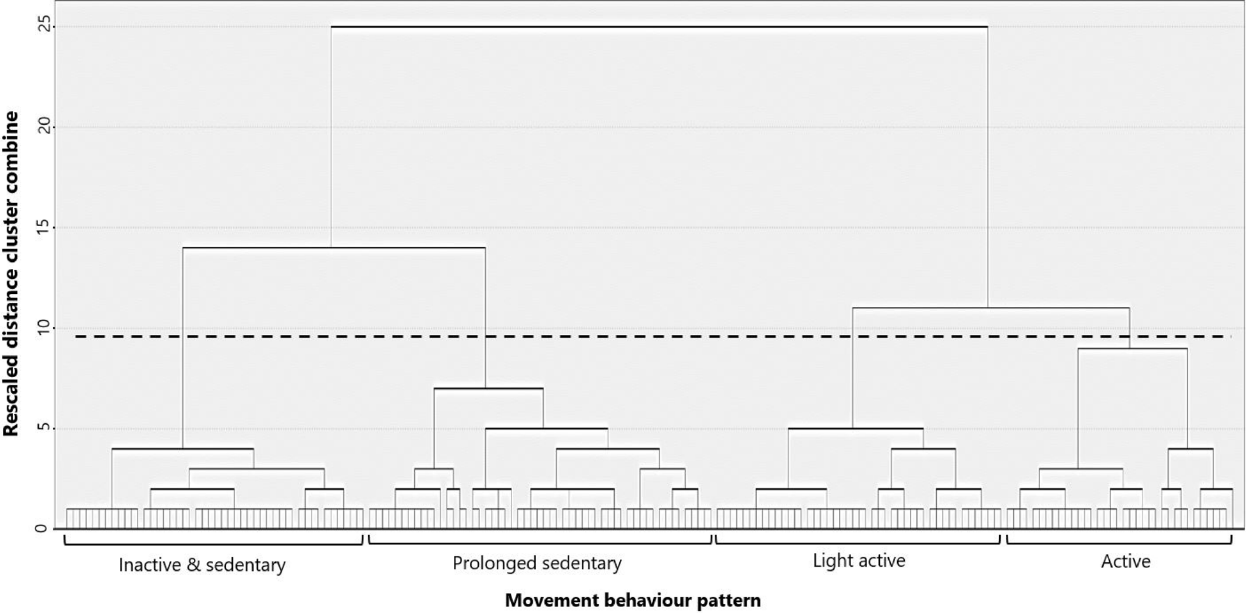


Note. Dendrogram illustrating the clustering of subjects, based on similarities in clustering variables. Cluster distance is expressed as Euclidean distance. The dashed line indicates the cutting level: the clustering procedure was stopped at the level where four subgroups were identified, later named "inactive & sedentary", "prolonged sedentary", "light active" and "active".
